# Case Report: Recurrent Hemiplegic Migraine Attacks Accompanied by Intractable Hypomagnesemia Due to a *de novo TRPM7* Gene Variant

**DOI:** 10.3389/fped.2022.880242

**Published:** 2022-05-31

**Authors:** Meifang Lei, Ping Wang, Hong Li, Xiaojun Liu, Jianbo Shu, Qianqian Zhang, Chunquan Cai, Dong Li, Yuqin Zhang

**Affiliations:** ^1^Department of Neurology, Tianjin Children’s Hospital (Tianjin University Children’s Hospital), Tianjin, China; ^2^Tianjin Pediatric Research Institute, Tianjin Children’s Hospital (Tianjin University Children’s Hospital), Tianjin, China; ^3^Tianjin Key Laboratory of Birth Defects for Prevention and Treatment, Tianjin, China; ^4^Department of Neurosurgery, Tianjin Children’s Hospital (Tianjin University Children’s Hospital), Tianjin, China

**Keywords:** *TRPM7* gene, *de novo* variant, ion channel, hemiplegic migraine, hypomagnesemia

## Abstract

Transient receptor potential melastatin 7 (TRPM7) is a ubiquitously expressed chanzyme comprised of a divalent cation channel permeable to calcium and magnesium and a cytosolic serine-threonine α-kinase domain. TRPM7 has a crucial role in magnesium ion homeostasis and anoxic neuronal death, which was identified as a potential non-glutamate target for hypoxic-ischemic neuronal injury. TRPM7 is implicated in ischemic stroke and hypomagnesemia in many studies, but it has not been associated with disease in the OMIM database. No clinical cases between *TRPM7* gene variants and hypomagnesemia have been reported, so far. One patient with recurrent hemiplegic migraine attacks accompanied by intractable hypomagnesemia was followed up at Tianjin Children’s Hospital from 2018 to 2021. We systematically summarized and analyzed the clinical manifestations, imaging features, and serum magnesium changes of the patient. Genetic analysis was performed by whole-exome sequencing and Sanger sequencing to infer the etiology of hemiplegic migraine attacks and hypomagnesemia in this patient. Gene sequencing revealed a novel heterozygous variant of the *TRPM7* gene (c.2998A>G, p. Met1000Val), which has not been reported previously; this is also a *de novo* variant that is not inherited from his parents. We described a novel variant p. Met1000Val (c.2998A>G) located in the transmembrane region of TRPM7 protein, which is possibly crucial for the normal function of the ion channel. Our study expands the variation spectrum of the *TRPM7* gene, highlights the importance of molecular genetic evaluation in patients with *TRPM7* gene deficiency, and demonstrates the causal relationship between *TRPM7* gene variants and disease manifestations.

## Introduction

The transient receptor potential melastatin (TRPM) family is an important cation channel family located in the cell membrane. The TRPM subfamily is composed of eight members including TRPM7, a widely expressed non-selective cation channel. TRPM7 is a dual function protein that plays an important role in many biological processes (1). The TRPM7 protein has four Melastatin Homologous Regions in the N-terminal, six transmembrane segments with a pore region located between the fifth and sixth transmembrane segment, and a serine/threonine-protein kinase domain in the C-terminal, which phosphorylates its substrates and regulates downstream target protein activity (2–4). The functional TRPM7 channel is a homologous tetramer, which has a high permeability to cations (2).

The molecular mechanism of Mg^2+^ transmembrane transport has made important advances following the discovery of the TRP superfamily. Schmitz and his colleagues found that TRPM7-deficient cells become magnesium ions deficiency, supplementation of extracellular magnesium ions can compensate for this defect, suggesting that TRPM7 has an important role in regulating cellular Mg^2+^ homeostasis (5). Another study showed that heterozygous TRPM7^Δ*kinase*^ mice exhibited signs of hypomagnesemia and impaired intestinal Mg^2+^ absorption, suggesting that TRPM7 is essential for the maintenance of Mg^2+^ homeostasis (6).

The N-methyl-D-aspartate glutamate receptors (NMDARs) mediated glutamate excitatory neurotoxicity and Ca^2+^ influx are important factors in neuronal injury during cerebral ischemia. However, clinical trials using anti-excitotoxic therapy to prevent stroke or traumatic brain damage did not achieve the desired efficacy, suggesting an additional mechanism of neuron injury induced by non-glutamate-dependent calcium overload may exist (7). TRPM7 channel was reported to cause neuronal death by non-glutamate-dependent calcium overload during ischemic hypoxia injury (7, 8). Another study confirmed the relationship between the TRPM7 channel and cerebral ischemic injury by establishing a rat model of cerebral ischemia (9).

No clinical cases of *TRPM7* gene variants causing hemiplegic migraine attacks and intractable hypomagnesemia has been reported so far. In this study, we systematically summarized the characteristics of clinical data and analyzed the *TRPM7* gene variant in a Chinese patient. We presented a *de novo* c.2998A>G (chr15:50891484) variant in *TRPM7* (NM_017672), which enriches the gene variation spectrum. Based on the correlation analysis between the patient’s clinical features and the TRPM7 protein function, we speculate that the c.2998A>G (p. Met1000Val) variant is responsible for the disease manifestations.

## Case Description

This patient was a 12-year-old boy, who was the first child born to non-consanguineous parents at term, without a history of perinatal hypoxic asphyxia. He was admitted to Tianjin Children’s Hospital in 2018 due to recurrent hemiplegic attacks for 7 years and headaches with right hemiplegia for 7 h; the right upper extremity was more paralyzed than the lower extremity. He presented with irritability, confusion, and slurred speech for 6 h before admission. He had recurrent hemiplegic attacks, since 5 years of age, mainly on the right side, with headache, dysarthria, salivation, drowsiness, and irritability, one of which was accompanied by contralateral ipsilateral hemianopia. Each attack lasted 4–5 min to 1–2 h, 2–5 times per year, and about 15 times in total. The shortest episode lasted 4–5 min and was relieved spontaneously, while the longest episode lasted 16 days to relieve, and the muscle strength and speech were normal during the interictal period. Rapid changes in temperature and infection can easily trigger the episodes, serum magnesium is monitored in each episode with the range of 0.36–0.56 mmol/L (normal range: 0.70–1 mmol/L). The concentration of C-reactive protein was elevated during two migraine attacks with a febrile respiratory infection, and it was normal when non-infective migraine occurs. The blood pressure of the patient was normal. Neurological examination showed irritability, confusion, and slurred speech. Nuchal rigidity. He was unable to complete ordered movements. The muscle tension of his right upper and lower limbs was low, proximal muscle strength of right upper limbs was grade II, distal muscle strength was grade III, and muscle strength of right lower limbs was grade III^+^. Bilateral knee-tendon reflex and Achilles tendon reflex at (++), while Bilateral Babinski signs at (+).

**FIGURE 1:**
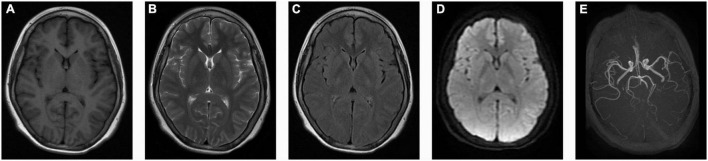
No abnormalities in the patient’s brain MRI and magnetic resonance angiography (MRA). **(A)** Axial T1-weighted images (T1WI); **(B)** Axial T2-weighted images (T2WI); **(C)** Axial T2 fluid attenuated inversion recovery sequence images (Flair); **(D)** Diffusion-weighted imaging (DWI); **(E)** Magnetic resonance angiography (MRA) images.

**FIGURE 2:**
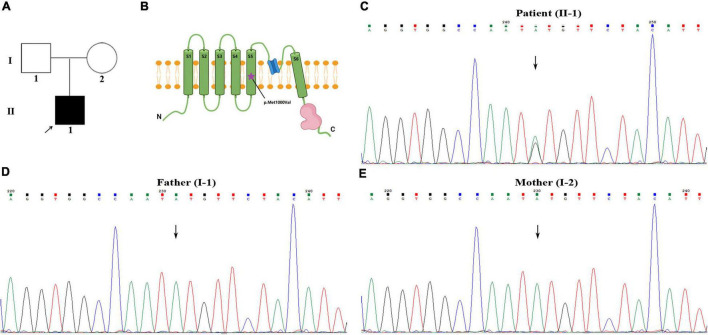
Identification of a *de novo* heterozygous variant of c.2998A>G in *TRPM7* gene. **(A)** Pedigree chart in this family. **(B)** The schematic diagram of TRPM7 protein molecular structure, the purple star indicates the variant is located in the fifth transmembrane segment. **(C–E)** Patient with c.2998A>G variant, his parents with normal genotype.

The cerebrospinal fluid (CSF) pressure was elevated to 285 mmH_2_O (normal range: 80–180 mmH_2_O, 1 mmH_2_O = 0.0098 kPa) when the headache was still present. The blood and CSF analysis of autoimmune encephalitis-related antibodies, oligoclonal bands, and paraneoplastic antibodies were negative. Electroencephalogram (EEG) showed full conductance diffuse of 1–3 HZ δ activity with left-right symmetry. Spinal cord and brain MRI and magnetic resonance angiography (MRA) showed no abnormalities (Figure 1). Electroneurogram is normal. The concentration of serum sodium and chlorine were slightly below the normal range when he was first admitted to the hospital, which returned to normal after sodium chloride supplementation. Serum magnesium was monitored for the patient with the range of 0.41–0.54 mmol/L (normal range: 0.70–1 mmol/L) during hospitalization. The random urine electrolyte test detected the concentration of magnesium (2.4 mmol/L), calcium (1.1 mmol/L), sodium (36.7 mmol/L), potassium (12.54 mmol/L), and chloride (31.7 mmol/L). Urinary magnesium excretion fraction (FEMg) was 9.7% (normal range: 2–4%). Serum parathyroid hormones are normal. No abnormality was found in blood and urine screening for genetic metabolic disease. The pedigree chart in this family was shown in Figure 2A.

**FIGURE 3:**
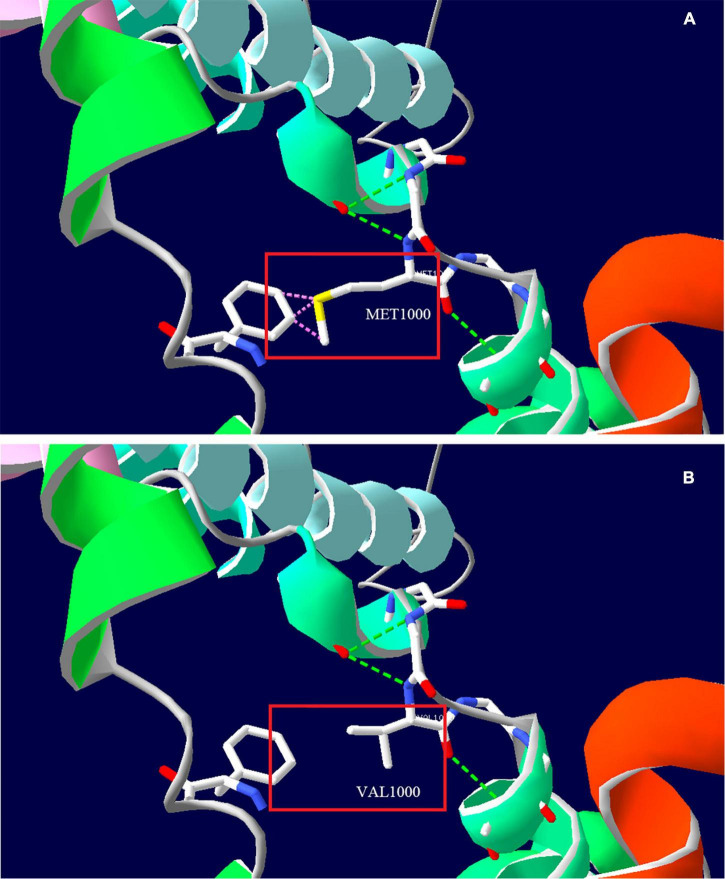
Three-dimensional structure prediction model of TRPM7 protein by SWISS-MODEL and Swiss-Pdb Viewer. **(A)** Amino acid side chain structure of the wild type (Met1000); **(B)** Amino acid side chain structure of the mutant type (Val1000). The pink dotted line indicates that the wild type may collide with the adjacent benzene ring (Phe1096), and there is no collision after mutation, the green dotted line indicates the hydrogen bond.

Whole exome sequencing (WES) was performed to investigate the genetic etiology. The results ruled out that *PRRT2*, *CACNA1A*, *ATP1A2*, *SCN1A*, and other common genes are known to cause hemiplegic migraine, while a heterozygous variant c.2998A>G (p. Met1000Val) was detected in *TRPM7* gene of this patient. This site is a *de novo* variant that is not inherited from his parents after Sanger sequencing verification (Figures 2C–E); it was also a novel variant, which is absent in gnomAD, 1,000 genomes, and ExAC databases. The functional prediction of this site was performed by Mutation Taster and sorting intolerant from tolerant (SIFT), it was predicted to be a deleterious variant. Based on ACMG (American College of Medical Genetics and Genomics) guidelines, the evidence of this site was PS2+PM2+PP3, which was classified as likely pathogenic. This site is located in the fifth transmembrane segment (Figure 2B), and the substitution of methionine (MET) to valine (VAL) results in increased hydrophobicity of amino acids. The three-dimensional structural prediction model predicted that the side chain structure of TRPM7 was changed, performed by SWISS-MODEL and Swiss-Pdb Viewer (Figure 3).

**FIGURE 4:**
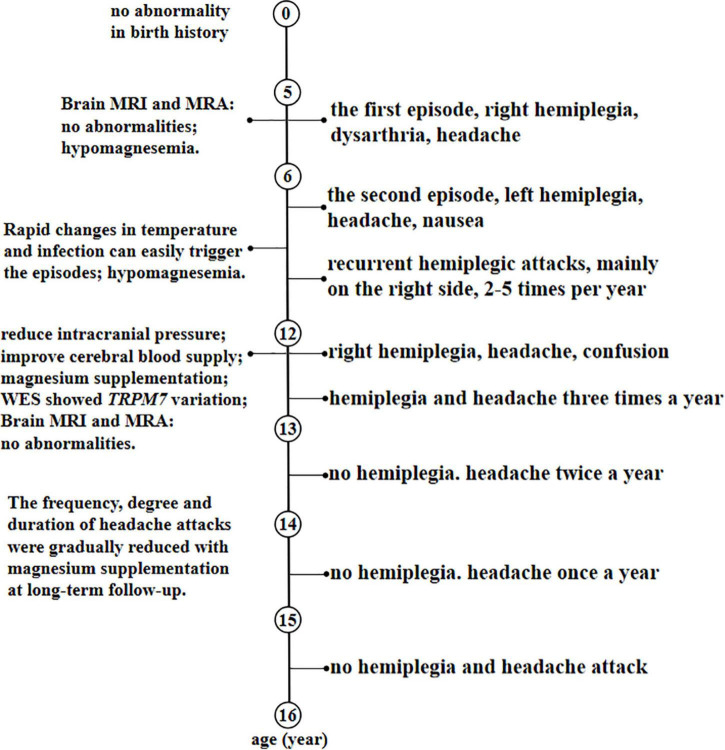
A timeline with relevant data from the patient episode of care.

Symptomatic treatment was given to the patient during hospitalization by reducing intracranial pressure, improving cerebral blood supply and intramuscular injection of 25% magnesium sulfate of 2.5 g/day. He was discharged from the hospital with normal muscle strength in all four limbs. He was given potassium and magnesium aspartate tablets with the amount of magnesium ion 70.8–106.2 mg/day since discharge for 6 months. Then, he changed to take magnesium carbonate effervescent tablets 1 tablet/day (equivalent to magnesium ion 57 mg) and was gradually increased to take 4 tablets/day (the dosage was not increased due to diarrhea), but the serum magnesium was stubbornly below normal range at long-term follow-up. Despite this, the frequency, degree, and duration of headache attacks were gradually reduced, and the symptoms changed from the original electric drill-like pain to the current throbbing pain, which showed that magnesium administration is effective in relieving symptoms. No hemiplegia attacks occurred in the past 2 years, and no headache occurred again in the past 1 year. He is 16 years old and attains developmental milestones with normal psychomotor development. The timeline with relevant data from the patient episode of care was shown in Figure 4.

## Discussion

The TRPM7 has not been associated with diseases in the OMIM database despite its role in diverse physiological and pathological processes, only with susceptibility to Amyotrophic lateral sclerosis-parkinsonism/dementia complex (10–12). No clinical cases of *TRPM7* gene variants causing hemiplegic migraine attacks and intractable hypomagnesemia have been reported, so far. TRPM7 channel is a non-selective cation channel that is permeable to divalent cations such as Ca^2+^, Mg^2+^, Zn^2+^, and monovalent cations such as K^+^. Gene variants in *TRPM6*, which is highly homologous to *TRPM7*, lead to hypomagnesemia with secondary hypocalcemia (13). TRPM6/7 heteromeric channels are likely to play an important role in intestinal and renal magnesium transportation (14). Thus, we speculated that TRPM7 may act alone or in combination with TRPM6 to regulate serum magnesium concentration in patients with hypomagnesemia.

In almost all tissues, TRPM7 is expressed. Vascular-expressed TRPM7 is regulated by the changes of vasoactive agents, pressure, stretch, and osmotic changes, which may be a novel mechanotransducer. As a signaling kinase, TRPM7 is also involved in vascular smooth muscle cell growth, apoptosis, adhesion, and contraction, which play important roles in many vascular diseases (15). Furthermore, the TRPM7 channel has been shown to cause neuronal injury and death through non-glutamate-dependent calcium overload during ischemic-hypoxic injury (7–9).

This study involved a case with complex clinical manifestations. A 12-year-old boy had recurrent hemiparesis and headache since the age of 5, with or without dysarthria, irritability, and drowsiness, and no abnormalities on brain MRI, which met the clinical diagnostic criteria for hemiplegic migraine (16) (no family history, sporadic case). He also presented persistent hypomagnesemia. WES results suggested a *de novo* variant of c.2998A>G (p. Met1000Val) in the *TRPM7* gene, which is located in the fifth transmembrane segment. The abnormal amino acid replacement in this site may result in impaired ion channel function, thus, resulting in dysfunction of renal and intestinal (re)absorption of magnesium. Notably, elevated urinary magnesium excretion fraction was observed in this patient indicating renal loss of magnesium, which may result in severe hypomagnesemia. So, we speculated that protein dysfunction due to the *TRPM7* gene variant was the cause of hypomagnesemia in this patient.

Hypomagnesemia induces sustained vasoconstriction without a transient vasodilatory phase, especially in the presence of endothelial damage (15). Variants in the *TRPM7* gene may lead to impaired vascular remodeling, and the concomitant presence of hypomagnesemia causes sustained vasoconstriction, which may be the cause of hemiplegic migraine attacks in the patient. Genes reported to be associated with hemiplegic migraine include *CACNA1A*, *ATP1A2*, *PRRT2*, and *SCN1A*, our study linked *TRPM7* gene variants to hemiplegic migraine for the first time, which may enrich the gene profile of this disorder.

Based on the clinical and biochemical characteristics, long-time follow-up, and treatment effect, we hypothesized that the recurrent hemiplegic migraine attacks and intractable hypomagnesemia of this patient are associated with the *TRPM7* gene variant. The patient is currently treated with magnesium supplementation and oral calcium channel blockers with good follow-up. Further functional validation experiments can help to confirm the relationship between the gene variant and protein function. We report this case in the hope that more peers will pay attention to the *TRPM7* gene variant and its related diseases.

## Data Availability Statement

The original contributions presented in the study are included in the article/supplementary material, further inquiries can be directed to the corresponding author/s.

## Ethics Statement

The studies involving human participants were reviewed and approved by the Medical Ethics Committee of Tianjin Children’s Hospital. Written informed consent to participate in this study was provided by the participants’ legal guardian/next of kin.

## Author Contributions

ML was involved in the design of the study and writing of the manuscript. PW contributed to the conception and revised the manuscript. HL contributed to the interpretation of the results and revised the manuscript. XL and JS participated in bioinformatics analysis and genetic analysis of the *TRPM7* gene. QZ contributed to clinical data collection. CC, DL, and YZ provided clinical diagnosis and genetic counseling. All authors read and approved the final manuscript.

## Conflict of Interest

The authors declare that the research was conducted in the absence of any commercial or financial relationships that could be construed as a potential conflict of interest.

## Publisher’s Note

All claims expressed in this article are solely those of the authors and do not necessarily represent those of their affiliated organizations, or those of the publisher, the editors and the reviewers. Any product that may be evaluated in this article, or claim that may be made by its manufacturer, is not guaranteed or endorsed by the publisher.
